# Is the *COL5A1* rs12722 Gene Polymorphism Associated with Running Economy?

**DOI:** 10.1371/journal.pone.0106581

**Published:** 2014-09-04

**Authors:** Rômulo Bertuzzi, Leonardo A. Pasqua, Salomão Bueno, Adriano Eduardo Lima-Silva, Monique Matsuda, Monica Marquezini, Paulo H. Saldiva

**Affiliations:** 1 Laboratory of Experimental Air Pollution, Department of Pathology, School of Medicine, University of São Paulo, São Paulo, Brazil; 2 Endurance Sports Research Group, School of Physical Education and Sport, University of São Paulo, São Paulo, Brazil; 3 Sports Science Research Group, Academic Center of Vitoria, Federal University of Pernambuco, Vitoria de Santo Antão, Brazil; 4 Faculty of Medicine, University of São Paulo, São Paulo, Brazil; University of Eastern Finland, Finland

## Abstract

The *COL5A1* rs12722 polymorphism is considered to be a novel genetic marker for endurance running performance. It has been postulated that *COL5A1* rs12722 may influence the elasticity of tendons and the energetic cost of running. To date, there are no experimental data in the literature supporting the relationship between range of motion, running economy, and the *COL5A1* rs12722 gene polymorphism. Therefore, the main purpose of the current study was to analyze the influence of the *COL5A1*rs12722 polymorphism on running economy and range of motion. One hundred and fifty (n = 150) physically active young men performed the following tests: a) a maximal incremental treadmill test, b) two constant-speed running tests (10 km•h^−1^ and 12 km•h^−1^) to determine the running economy, and c) a sit-and-reach test to determine the range of motion. All of the subjects were genotyped for the *COL5A1* rs12722 single-nucleotide polymorphism. The genotype frequencies were TT = 27.9%, CT = 55.8%, and CC = 16.3%. There were no significant differences between *COL5A1* genotypes for running economy measured at 10 km•h^−1^ (p = 0.232) and 12 km•h^−1^ (p = 0.259). Similarly, there were no significant differences between *COL5A1* genotypes for range of motion (p = 0.337). These findings suggest that the previous relationship reported between *COL5A1* rs12722 genotypes and running endurance performance might not be mediated by the energetic cost of running.

## Introduction

It is well known that athletic performance is dependent on a multifactorial phenotype resulting from a complex interaction between environmental [1], [2] and genetic [3], [4] factors. In the past few years, researchers have analyzed the influence of some genes that encode proteins that are involved in metabolic pathways [5] or skeletal muscle structure [4], [6], which are important to athletic performance. More recently, Posthumus et al. [7] reported that endurance running performance is associated with a gene, named *COL5A1,* which encodes a structural protein of the extracellular matrix. These authors observed that the TT genotype was overrepresented in runners with faster performances during the 42.2-km running portion of the Ironman triathlon, suggesting that*COL5A1* might be a novel genetic marker for endurance running performance.

It is believed that the relationship between *COL5A1* and endurance running performance might be mediated by the energy storage capacity of the connective tissues [7],[8]. *COL5A1* encodes the α1 (V) chain of type V collagen [9], which plays a crucial role in the regulation of the size and configuration of other abundant fibrillar collagens supporting many tissues in the body, such as tendons, ligaments, and muscles [10]. A mutation in the COL5A1 gene, which causes haploinsufficiency, results in a 50% reduction of type V collagen, and leads to poorly organized fibrils, decreased tensile strength, and reduced stiffness of connective [9]. It has been demonstrated that a common C to T single nucleotide polymorphism within the COL5A1 3′ untranslated region (rs12722) may alter COL5A1 mRNA stability and thereby reduce the production of type V collagen [11]. Thus, it was hypothesised that individuals with a TT genotype of this variant would have increased type V collagen production and thus favorably altered mechanical properties of tendons, which enhances endurance running ability [7], [8].

Running economy (RE), which has been defined as the energy cost or oxygen uptake for a given submaximal running speed [12], [13], is considered to be an important determinant of endurance performance [14], [15]. It has been indicated that the inter-individual variation in RE is approximately 10–25% between homogeneous subjects for maximal oxygen uptake 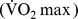 [15]. The cause of this variation is not well understood, but it might be partially explained by the energy storage capacity of the tendons [14]. Indeed, elastic energy is generated by the passive stretch of the muscle elastic elements, and this energy is converted to kinetic energy free of metabolic cost during a running bout [16]. Thus, the most economical runners have a higher compliance of the tendons and aponeuroses compared to their less economical counterparts [14]. Because of this reported association between RE and the energy storage capacity of the tendons, it has been proposed that individuals with a TT genotype would have less extensible tendon structures, resulting in a lower range of motion (ROM) and greater RE when compared with individuals with at least one copy of the C allele [8]. However, to date there are no experimental data in the literature supporting this relationship among RE, ROM, and the *COL5A1* rs12722 gene polymorphism. Consequently, it is difficult to determine whether the association previously reported between the *COL5A1* gene and endurance performance was due to a reduced running energy cost.

Therefore, the main objective of the present study was to analyze the influence of the *COL5A1* gene polymorphism on RE. In light of past studies that reported a relation between the *COL5A1* gene (rs12722) and endurance running performance [7], [8], it was hypothesized that individuals with a TT genotype would have a greater RE and a lower ROM when compared to individuals with the TC and CC genotypes.

## Materials and Methods

### Participants

One hundred and fifty (n = 150) physically active men (age 25.2±4.0 years, body mass 77.8±13.9 kg, height 173.9±21.4 cm, and body fat 13.3±4.2%) volunteered to participate in this study. All subjects were medication-free, nonsmokers, and free of neuromuscular disorders and cardiovascular dysfunctions. The subjects had been involved in recreational sports in the past year, but they had not engaged in flexibility and strength training for at least six months before the study. The participants received a verbal explanation about the possible benefits, risks, and discomforts associated with the study and signed a written informed consent before participation in the study. The procedures were in accordance with the Helsinki declaration of 1975, and the study was approved by the Ethics Committee for Human Studies of the School of Physical Education and Sport of the University of São Paulo.

### Experimental design

All of the subjects were required to visit the laboratory on three occasions separated by at least 72 h over a 2-week period. In the first visit, the subjects were asked to perform mouthwashes for genomic DNA extraction, to perform a sit-and-reach test for ROM measurement, and to fill out a short version of the physical activity questionnaire (IPAQ-short version) to estimate their physical activity levels. In the second visit, anthropometric measurements (height, body mass, and body composition) were recorded and a maximal incremental treadmill test was performed. A constant-speed, treadmill running familiarization test was conducted at the end of the first and second visits after a 20-min passive recovery. In the third session, the subjects performed two submaximal constant-speed tests. All of the tests were performed at the same time of day in a controlled-temperature room (20–24°C) and 2–3 h after the last meal. All of the subjects were asked to refrain from any exhaustive or unaccustomed exercise for 48 h preceding the test. They also were instructed to wear standard running shoes and asked from taking off nutritional supplements six months before the experimental period.

### Anthropometric measurements

All of the anthropometric measurements were made according to the procedures described by Norton and Olds [17]. The subjects were weighed using an electronic scale to the nearest 0.1 kg. Height was measured with a stadiometer to the nearest 0.1 cm. Skinfold thickness was measured at seven sites (chest, axilla, triceps, subscapular, abdominal, suprailiac, and thigh) with a Harpenden caliper (West Sussex, UK) to the nearest 0.2 mm. The seven skinfold thickness values were obtained on the right side of the body in a serial fashion, and the median of three values was used for data analysis. When the difference between these three values was higher than 10%, a fourth measurement was obtained. All measurements were made by the same experienced investigator. Body density was estimated using the generalized equation of Jackson and Pollock [18], and body fat was estimated using the equation of Brozek et al. [19].

### Maximal incremental treadmill test

The subjects performed a maximal incremental test on a motor-driven treadmill (model TK35, CEFISE, Nova Odessa) to determine maximal oxygen uptake 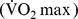. After a 3-min warm-up at 8 km·h^−1^, the velocity was increased by 1 km·h^−1^ every minute until exhaustion. The participants received strong verbal encouragement to ensure attainment of maximal values. Gas exchange was measured breath-by-breath using a gas analyzer (Cortex Metalyzer 3B, Cortex Biophysik, Leipzig, Germany) and was subsequently averaged over 20 s intervals throughout the test. Before each test, the gas analyzer was calibrated according to the recommendations of the manufacturer. Maximal heart rate (HR_MAX_) was defined as the highest value obtained at the end of the test. Blood samples (25 µl) were collected from the ear lobe one, three, and five minutes after the test to determine the peak blood lactate. Lactate concentrations were measured spectrophotometrically (EONC, Biotek Instruments, USA) using a wavelength of 546 nm. 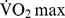 was determined when two or more of the following criteria were met: an increase in oxygen uptake of less than 2.1 ml·kg^−1^·min^−1^ between two consecutive stages, a respiratory exchange ratio greater than 1.1, and a ±10 bpm of the predicted maximal heart rate (i.e., 220-age) [20].

### Running economy

The subjects performed two constant-speed running tests on a treadmill to determine RE. Because it has been reported that a subject who is economical at a given speed will not be necessarily economical at other speeds [21], we measured the RE at10 km·h^−1^ and 12 km·h^−1^ speeds. These intensities corresponded to 78.8±6.7% and 89.7±7.9% of the 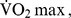 respectively. Due the different percentage values required of the 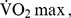 it was assumed that these intensities represented the RE at low (RE_LW_ = 10 km·h^−1^) and high (RE_HG_ = 12 km·h^−1^) intensities. Before the tests, the participants performed a standardized warm-up consisting of a 5 min run at 8 km·h^−1^ followed by a 5-min of passive recovery. RE was determined by measuring breath-by-breath oxygen uptake for 10 minutes at each running speed. RE was defined by averaging the oxygen uptake values during the last 20 s for each running speed. Recovery time between these two constant-speeds running tests was 10 min.

### Range of motion

Range of motion (ROM) was measured using a sit-and-reach test [22], [23]. This test has been used in previous studies investigating the effects of the *COL5A1* genotypes on lower limb flexibility because it represents an indirect measure of both hamstring musculotendinous unit length and lumbar ROM [25], [26]. The subjects sat with their bare feet pressed against the sit-and-reach box. The knees were extended and the right hand was positioned over the left. Then, the subjects were asked to push a ruler transversally located over the box as far as possible on the fourth bobbing movement. Three trials were performed, and the best trial was used for statistical analysis.

### Physical activity level determination

Because the RE may be influenced by the training status of the subjects [27], the short version of the International Physical Activity Questionnaire (IPAQ-SV) was used to identify the physical activity level of the participants. This questionnaire was developed with a multi-cultural adaptation and is considered to be one of the most widely utilized instruments due to the quickness of data collection, low operating cost, and non-invasive characteristics [28]. In addition, it has recently been shown that IPAQ-SV outcomes are associated with flexibility and cardiorespiratory fitness levels in healthy men [29]. The participants answered the questionnaire in a classroom setting after a detailed description of the IPAQ-SV. An assistant remained in the classroom setting for eventual doubts. The major aim of the IPAQ-SV is to sum up walking, moderate and vigorous PA and to generate a total PA score for weekly expenditure, expressed in metabolic equivalent task units (METs min/wk). We used the following recommended METs min/wk estimates of the IPAQ-SF: walking PA = 3.3 METs min/wk, moderate PA = 4.0 METs min/wk, vigorous PA = 8.0 METs min/wk. The total PA was calculated assuming: 3.3×walking PA+4.0×moderate PA+8.0×vigorous PA. The PA level was classified as low, moderate or high. Low activity represented individuals who do not meet the criteria for moderate and vigorous intensity categories (<599 METs min/wk). Moderate activity represented moderate or vigorous intensity activities achieving a minimum of at least 600 METs min/wk. High activity represented participants achieving a minimum of at least 3000 METs min/wk (http://www.ipaq.ki.se/scoring.htm).

### Genotype assessment

Cells from the mouthwashes were submitted to an overnight digestion with proteinase K. Nucleotides were separated from the cellular debris by density gradient centrifugation using chloroform. Genomic DNA was then precipitated with isopropyl alcohol, isolated by centrifugation and resuspended in TE buffer. DNA quantification was performed using a spectrophotometer (NanoDrop, ND 2000, USA), and the concentration was adjusted to 1 µg/µL for subsequent storage in −20°C. *COL5A1* rs12722 gene polymorphisms were determined by conventional 2-primer PCR (F: 5′-GCAGTCAGCAGCGTGGGTCTGGTTATCT-3′ and R: 5′-TTTGGGGTGGCACTTGCAGCACT GGTCG-3′). This assay resulted in the amplification of a 637-bp fragment of the *COL5A1* gene that includes the polymorphic region. The reaction conditions were as follow: initial hold at 94°C for 3 min, 35 cycles of denaturation at 94°C for 60 s, annealing at 53°C for 60 s and extension at 72°C for 60 s, and a final extension step of 8 min at 72°C. The amplified fragment was subsequently digested using B*st*UI (New England, Biolabs, Beverly, MA, USA), following the supplier’s recommendations. The digested products were then separated on a 3% agarose gel. To ensure proper internal control, for each batch of analysis, we used positive and negative controls from different DNA aliquots that were previously genotyped by the same method, according to recent recommendations for replicating genotype-phenotype association studies [30]. The restriction fragment length polymorphism (RFLP) results were scored by three experienced and independent investigators who were blinded to the participant’s data.

### Statistical analysis

Data normality was assessed using the Kolmogorov-Smirnov test, and all of the variables showed a normal distribution. The results are reported as means and standard deviations (±SD). A X^2^ test was used to verify that the genotype frequencies were in Hardy-Weinberg equilibrium. The effects of the *COL5A1* genotypes in the analyzed variables were tested using a one-way analysis of variance (ANOVA). The significance level was set at p<0.05. All of the statistical analyses were performed using Statistica 8 (StataSoft Inc., Tulsa, OK, USA).

## Results

### Genotype distribution and sample characteristics

The genotype distribution attained the Hardy-Weinberg Equilibrium, as evidenced by the X^2^ test. The genotype frequencies were TT = 27.9%, CT = 55.8% and CC = 16.3%. The genotype distribution of the *COL5A1* rs12722 gene polymorphism was similar to the distribution reported in the public databases for Caucasian populations (http://opensnp.org/snps/rs12722). In accordance with the mean values of the IPAQ-SV outcomes, the subjects of the three groups were classified as moderately active [31]. There were no differences in age, anthropometric characteristics, or physical activity levels among the CC, CT, and TT genotypes of the *COL5A1* gene (p>0.05) (table 1).

**Table 1:** Anthropometric characteristics and physical activity levels of the subjects in the three genotype groups.

	CC (n = 24)	CT (n = 84)	TT (n = 42)	P values
Age (years)	25.7±3.7	21.3±4.2	23.8±5.0	0.873
Body mass (kg)	80.5±10.7	76.1±9.9	75.2±10.4	0.230
Height (cm)	177.8±5.4	172.6±6.5	174.2±5.8	0.243
Body fat (%)	14.3±3.8	13.2±4.2	13.9±4.6	0.584
IPAQ-SV (score)	1291±237	1356±289	1333±301	0.605

Data are means ± standard deviations. IPAQ-SV: short version of the international physical activity questionnaire. There were no differences between the groups.

### Maximal incremental, running economy, and range of motion tests

**Figure 1:**
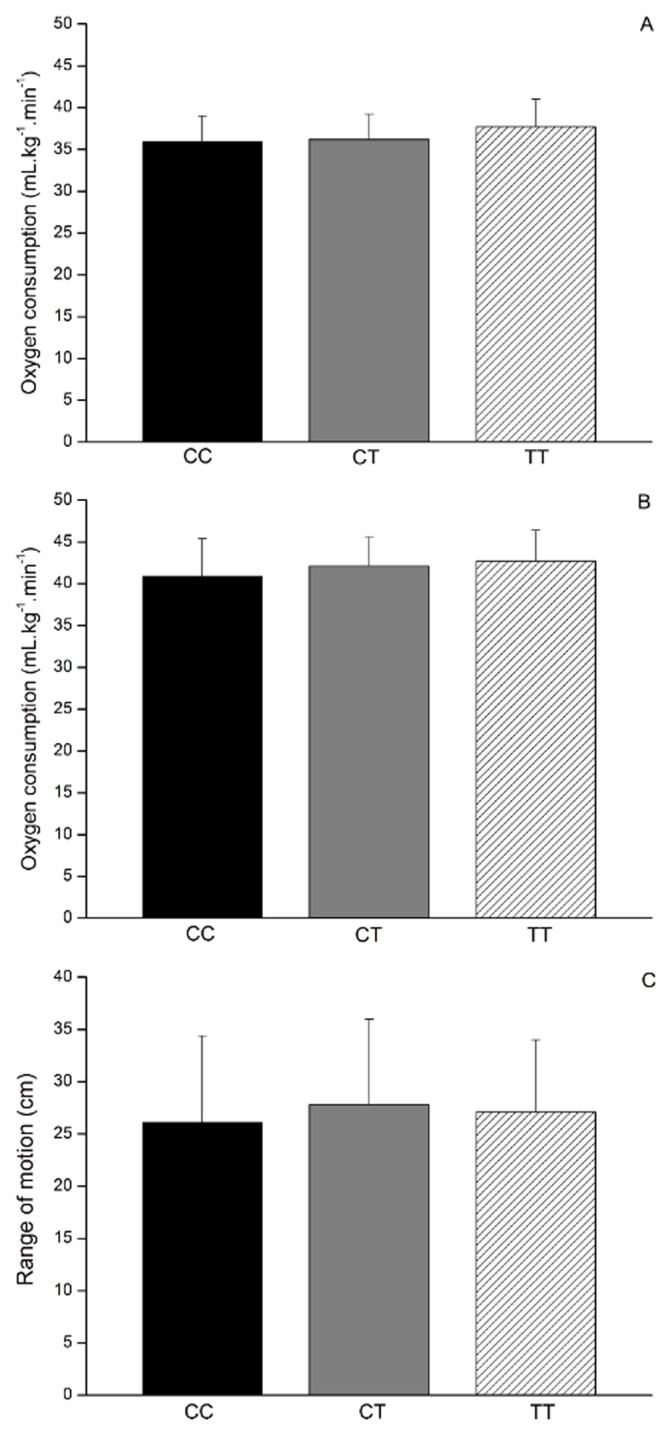
Running economy, range of motion, and *COL5A1* (rs12722) genotypes. Panel A: running economy at low intensity, Panel B: running economy at high intensity, Panel C: range of motion. There were no differences between the groups.

Table 2 shows the mean values of the physiological variables measured during the maximal incremental test. There were no significant differences in 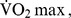 HR_MAX_, and [La]peak between individuals with different *COL5A1* genotypes (p>0.05). Figure 1 shows the results of the constant-speed running and sit-and-reach tests. The RE_LW_ (p = 0.232) (panel A), RE_HG_ (p = 0.259) (panel B), and ROM (p = 0.337) (panel C) were not statistical different between the CC (RE_LW_ = 35.97±3.18 ml·kg^−1^·min^−1^; RE_HG_ = 40.98±4.59 ml·kg^−1^·min^−1^; ROM = 26.14±8.34 cm), CT (RE_LW = _36.03±2.93 ml·kg^−1^·min^−1^; RE_HG_ = 41.80±3.36 ml·kg^−1^·min^−1^; ROM = 27.85±8.22 cm), and TT (RE_LW = _37.65±3.37 ml·kg^−1^·min^−1^; RE_HG = _42.66±3.79 ml·kg^−1^·min^−1^; ROM = 27.08±6.88 cm) *COL5A1* genotypes groups.

**Table 2:** Physiological variables measured during the maximal incremental test in the three genotype groups.

	CC (n = 24)	CT (n = 84)	TT (n = 42)	P values
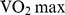 (mL.kg^−1^.min^−1^)	47.1±5.2	47.4±6.1	47.6±5.8	0.966
HR_MAX_ (bpm)	190±10	189±9	189±7	0.940
[La]peak(mmol.L^−1^)	9.9±3.7	9.4±3.9	9.6±4.1	0.781

Data are means ± standard deviations. 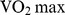: maximal oxygen uptake, HR_MAX_ = maximal heart rate, [La]peak: peak of blood lactate accumulation. There were no differences between the groups.

## Discussion

Previous findings suggested that the *COL5A1* gene might be a marker of endurance running performance [24], [7]. It has been speculated that the superior running ability of individuals with a TT genotype could be explained by a greater RE when compared with individuals with at least a C allele [8]. However, it is important to notice that these studies did not investigate the relationship between the energetic cost of running and *COL5A1*genotypes because the subjects did not perform constant-speed tests to measure the RE. To the best of our knowledge, this is the first study designed to analyze the relationship between a common C to T single-nucleotide polymorphism gene within *COL5A1* gene (rs12722) and RE. The major findings of this study show that the*COL5A1* genotypes were not associated with either RE or ROM.

It has been proposed that mechanical properties of connective tissues are responsible for converting elastic energy to kinetic energy free of metabolic cost [14]. It was demonstrated that more economical subjects have a higher contractile strength and compliance of the tendons and aponeuroses when compared with their less economical counterparts [14]. Some studies have suggested that the mechanical properties of tendons and ligaments, which largely consists of collagen fibrils, may be genetically determined [32], [10]. In particular, the genetic variation in the COL5A1 gene, which encodes type V collagen, may affect the mechanical properties of tendons and ligaments through altering the mechanical properties. It has previously been shown that the COL5A1 gene variant investigated in this study was associated with endurance running ability. Although the specific mechanisms remain largely unknown, we hypothesised in this study that this gene variant improves endurance running ability through an improving running economy. However, the present findings did not confirm the relationship between *COL5A1* genotypes and RE. Our results showed no significant differences in RE_LW_ between individuals with different *COL5A1* genotypes. This finding suggests that the superior performance observed in individuals with a TT genotype in the *COL5A1*gene (rs12722) cannot be explained by a lower energy cost during running at a low percentage of 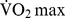.

In the present study, we considered that a subject who is economical at low relative running intensity might not be economical at high relative running intensity. Our results demonstrated that *COL5A1* genotypes were not associated with the energetic cost of running regardless of running intensity. An alternative explanation for the relationship between *COL5A1* genotypes and endurance running performance might be the ability to produce force rather than a reduced energetic cost. Recently, Kubo et al. [33] demonstrated that individuals with a T allele had a higher stiffness of the knee extensors compared to those individuals with at least one copy of the C allele. It was previously demonstrated that dynamic muscle actions are positively related to stiffness, possibly due a more effective force transmission from the contractile elements to the bone [34]. In turn, the ability to produce force has been considered an essential determinant of endurance performance without being necessarily related to RE [35], [36]. This occurs because the horizontal component of ground reaction force is fundamental for endurance runners to attain high-intensity running speeds [35]. Thus, individuals with a TT genotype may be able to maintain higher running speeds during long-distance events than their counterparts with a CT or CC genotype by applying greater forces to the ground. Nevertheless, this hypothesis should be analyzed with caution because we were unable to obtain ground reaction forces in the present study. Thus, further research is needed to examine the underlying mechanisms determining the relationship between *COL5A1* genotypes and endurance running performance.

It has also been postulated that *COL5A1* rs12722 genotypes could alter the elasticity of tendons [10] and contribute to explaining the inter-individual variation in ROM [36] additionally to other polymorphisms (*i.e. COL5A1* rs71746744) of this gene [37]. However, conflicting results were reported in the literature regarding the relationship between *COL5A1* gene polymorphisms and ROM. Some studies found that the CC genotype was overrepresented in individuals with greater ROM [24], while others found no relationship [25]. In the present study, the ROM values between individuals with CC, CT, and TT genotypes were not significantly different. This result is in agreement with the earlier findings of Brown et al. [25] who found that *COL5A1* genotypes in a South African cohort were not associated with ROM in young subjects (<35 years). However, these authors found a positive correlation between *COL5A1*genotypes and ROM in older subjects (>38 years old) [26]. It is important to note that besides our cohort consisting of younger subjects (25.2±4.0 years), a significant difference was not detected for age between individuals with different *COL5A1* genotypes. Taken together, these findings reinforce the suggestion that *COL5A1* rs12722 genotypes might interact with age for ROM in physically active subjects [26].

It is important to acknowledge some of the limitations of the present study. First, the subjects in the present investigate on were characterized as physically active, as evidenced by the IPAQ-SV outcomes. This would imply that our subjects might have a lower capacity for endurance exercise than the trained endurance runners that were previously studied [24], [7]. Thus, caution should be exercised in extrapolating these findings for highly-trained endurance runners. Second, the present study was conducted on a relatively small sample size. Therefore, our findings need to be confirmed in a larger cohort of subjects. On the other hand, it is important to notice that in the present study no differences for physical activity levels, age, and anthropometric measurements were observed among the three *COL5A1* (rs12722) genotypes (Table 1). In addition, our cohort was composed exclusively of men. This seems to be especially important because training status [38], age [39], body weight [27], and sex [36] all influence RE. Therefore, it is reasonable to assume that most of the potential confounding variables were controlled in the current study.

In conclusion, the results of the current study demonstrated that variants within the *COL5A1*gene were not associated with RE and ROM. This indicates that the previous relationship reported between *COL5A1* genotypes and endurance running performance may not be mediated by the energetic cost of running. Therefore, further studies are needed to examine the causal relationship between *COL5A1* gene and endurance running performance.
